# Effect of Bizhongxiao Decotion (BZXD) on Some Cytokines in Plasma of Rats with CII-induced Rheumatoid Arthritis

**Published:** 2005-06

**Authors:** An-yu Wang, Qing-hua Liang, Xu Luo, Tai-cheng Bao, Chun-yan Li, Jiang Cheng, Xiao-chun Liu

**Affiliations:** *Xiangya Hospital, Central South University, Changsha, China*

**Keywords:** bizhongxiao decotion, methotrexate, arthritis, rheumatoid, TNF-α, IL-1β

## Abstract

**Objective:**

To investigate the influence of bizhongxiao decoction (BZXD) which is a Traditional Chinese medicine for RA including, on the plasma TNF-α and IL-1β in rats with CII-induced arthritis (CIA) and explore the protective mechanism of BZXD in the treatment of rheumatoid arthritis.

**Methods:**

75 SD rats were divided into four groups randomly. Normal control group (n=5) not be treated any more. The CIA rat was established by subcutaneous injection with bovine II collagen (B II C) and complete Freund, s adjuvant (CFA) after 7d breeding. The CIA rats were divided into the CIA group (n=16), BZXD group (n=29) treated with BZXD and the MTX group (n=25) treated with methotrexate. All rats were killed after various intervals (25, 30, 35, 40, or 45d). At the end of each time interval, we collected the blood of each rat. To detect TNF-α and IL-1β in plasma with radio-immunity kit.

**Results:**

BIIC and CFA can be used to copying CIA model. The incidence of arthritis was 88%. The plasma TNF-α and IL-1β levels of CIA group, BZXD group and MTX group were notably higher than those of normal control group (*p*<0.05), moreover, the CIA group was higher than those of the MTX group and BZXD group at various interval (*p*<0.01). TNF-α and IL-1β rose step by step in CIA group but decreased in BZXD group and MTX group gradually. Moreover, in BZXD group were lower than those in MTX group (*p*<0.05).

**Conclusion:**

TNF-α and IL-1β play a very important role in the formation and development of RA. BZXD can notably decrease the plasma TNF-α and IL-1β levels, which was better than MTX.

## INTRODUCTION

Rheumatoid arthritis (RA) is particular with chronic and symmetric arthritis which get along with joint damage and matrix erosion. But its mechanism has not been clear. The recent research has demonstrated that TNF-α and IL-1β play key role in the disease of RA and made progress with matrix erosion. To explore the mechanism of the RA and the function of BZXD, we make the collagen-induced arthritis rat and treated with BZXD, then detect the level of TNF-α and IL-1β in plasma at various interval.

## MATERIALS AND METHODS

### Material

Of 75 SD rats (age: 45-50 days old, weight 150 ± 30 g), 37 female and 38 male of rats were selected for experiment which were purchased from Xiang Ya medical college experimental center, central southern university. Bovine II Collagen was purchased from the company of Sigma. Freund’s complete adjuvant (FCA) was bought from the company of Gbica. TNF-α and IL-1β radio-immunity kits were bought from the East Asia immunity center of Beijing.

### Methods

Dividing experimental animals. Seventy-five SD rats are divided into 4 groups randomly: 1) Normal control group (N=5); 2) CIA group (N=16); 3) MTX group (N=25); 4) BZXD group (N=29).

Rat model established (1). Grinded collagen type-II with FCA, and then injecting the compound 100 μl into the rat tail skin for each one. For enhancing the immunity effect, injecting again according to the above method after 21 days.

**Treatment.** 7 days after immunity, rats in BZXD group and MTX group were treated respectively which in BZXD group was treated with BZXD 10ml.kg-1twice every day (the usage equal to 3 times of 70 kg person) and in MTX group was treated with MTX 2.7mg.kg - lonce every week (the usage equal to 3 times of 70 kg person). Rats in normal control group not be treated any more.

**Arthritis symptom evaluation (2).** Arthritis index which is according to the red and swollen degree in joint was adopted to evaluate symptom. 0 cents: no arthritis; 1 cent: light swelling or few red spot; 2 cents: middle degree swelling in joint; 3 cents: joint swollen badly and can’t sustain themselves. The joint index more high, the joint symptom more serious.

**Method of obtaining tissues.** Rats in normal group are killed at 25d. Those rats in CIA group, ZXD group and MTX group were killed at 25d, 30d, 35d, 40d, 45d respectively. After using anticoagulantand and separating plasma with EDTA, these specimens were protected in -20°C for use. Above process go according to the kits introduction.

### Statistical analysis

All the data were analysised by using statistical software package (spss 11.0) and were manifested by mean and standard deviation ($\bar{X}$ ± s). Different groups were compared with analysis of variance and t-test.

## RESULTS

### The general condition

CIA rats eat less and weigh lighter than those in the other three groups. Moreover, they have dry hair. After the first time immunity, the partial rats have light swelling and red spot at joint. Ten days later, the arthritis get more bad. After enhancing immunity at 21d, the symptom get serious more and more. CIA rats swellen in ankles and toes symmetry. But in the BZXD group and the MTX group, those rats have joint swelling and inflammation alleviates gradually from 30d while the CIA group have the highest arthritis index.

### The result of the arthritis index

There were no differences among these three groups before 14 d. Following the time advancing, the arthritis index increase gradually. The symptom of those rats in BZXD group and MTX group rise to the peak at 21 d and 25 d respectively, which was lower than those in CIA group. To 30 d, the arthritis index decline.

**Plasma TNF-α and IL-1β levels (Table 1) (Table 2)**

**Table 1:** Plasma TNF-α level at different time (ng.L^-1^)

Groups	n	25d	30d	35d	40d	45d

Normal group	5	0.373 ± 1.287				
CIA group	16	51.891 ± 6.023^a^	57.878 ± 4.128^a^	59.982 ± 2.491^a^	63.220 ± 7.983^a^	64.510 ± 3.504^a^
MTX group	25	41.096 ± 6.866^a^^c^	37.061 ± 3.890^a^^b^	36.065 ± 6.210^a^^b^	35.733 ± 5.112^a^^b^	32.675 ± 7.405^a^^b^
BZXD group	29	32.684 ± 8.757^a^^b^^d^	28.880 ± 5.791^a^^b^^d^	28.257 ± 4.931^a^^b^^d^	26.608 ± 8.193^a^^b^^d^	26.322 ± 5.408^a^^b^^d^

a Compare with normal group *p*<0.01;

b Compare with CIA group *p*<0.01 *p*<0.05 --compare with MTX group p<0.05.

**Table 2:** Plasma IL-1β level at different time (ng.ml^-1^)

Groups	n	25d	30d	35d	40d	45d

Normal group	5	1.3936 ± 0.2046				
CIA group	16	2.3542 ± 0.1724^a^	2.4880 ± 0.1660^a^	2.5535 ± 0.1701^a^	2.6344 ± 0.1665^a^	2.7103 ± 0.1670^a^
MTX group	25	2.3061 ± 0.2351^a^	2.1070 ± 0.2285^a^^b^	1.9271 ± 0.2155^a^^b^	1.8063 ± 0.2260^a^^b^	1.7015 ± 0.2141^a^^b^
BZXD group	29	2.1440 ± 0.2210^a^^b^^c^	1.8733 ± 0.2185^a^^b^^c^	1.7131 ± 0.2201^a^^b^^c^	1.6074 ± 0.2167^a^^b^^c^	1.4920 ± 0.2199^a^^b^^c^

a Compare with normal group *p*<0.05;

b Compare with CIAl group *p*<0.01;

c Compare with MTX group<0.05.

## DISCUSSIONS

The RA held chronic and symmetry arthritis as primary expression which based on the synovitis. Its cause and mechanism are unclear yet. With the cell molecular biology and the immunology developing and the RA animal model established, these information can help to find mechanism such as cytokines, TH1/ TH2 cell balanced, cell apoptosis, sex hormone, proto-oncogene which are of great importance to the outbreak of RA. Among numerous cytokines, people have confirmed that the IL-1, TNF-α played a key role in arthritis progression (3, 4). TNF-α is an important regular factor in inflammation and immunity response, which can stimulate the synoviocyte and cartilage cells to synthesize the PGE2 and collagenase causing synovium and cartilage destruction in joint. TNF-α can stimulate itself synthesization as well as those of IL-1, IL-6, IL-8. It is one of the most important factors in the cytokine network. IL-1 includes IL-1α and IL-1β which is mainly secreted by macrophage, the endothelial cells and lymphoid cells. It might to induce a series of inflammation response including fever, synthesization of the acute period protein and influence of the local cartilage and the matrix metabolism. There was a study showed that TNF-α blocker is valid in the early arthritis, but the treatment of the anti- IL-1 can prevent the arthritis from developing continuously and the cartilage destruction (5, 6). In rabbit arthritis model, using adenovirus carrier to load the IL-1 solube receptor into knee joint can reduce the cartilage erosion and the leucocyte infiltration (7). IL-13 have also anti-inflammation activity because of suppressing IL-1β and TNF-α expression (8). Wim van den Berg (9) pointed that TNF-α caused early joint swelling in RA and IL-1β combining with the immune complex lead to the cartilage erosion. TNF blockage was effective in the early period, and following anti-IL-1 treatment was effective on the blockage of arthritis and joint destruction, he said. At the same time, IL-1 affect TNF-α mutually which aggravate the disease. TNF-α which can induce IL-1 production is important at the onset of RA. And based on this view, he pointed that it is necessary to block IL-1 and TNF-α in the treatment of RA. Observing in this experiment, the joint swelling and inflammation was obvious after established the mold 25d. TNF-α and IL-1β rise remarkably with the disease progress which indicated these two cytokines play the key role in RA pathologic process. Following TNF-α and IL-1β declined, the symptom of the red and swelling alleviated in joint after treated with BZXD and MTX. Which coincided with the above report.

The BZXD is a Traditional Chinese medicine for active period RA which can promote blood circulation and remove blood stasis, clear away heat and toxic materials, induce diuresis to reduce edema and dispel pathogenic mind and remove obstruction in the channels. BZXD can relieve symptom such as morning stiffness, joint ache, joint swelling and so on in the treatment with active period RA. All these role of BZXD were better than those of MTX. At the same time, the BZXD still has the anti- immunity and regulating the sub-cluster in T cells which resemble with the MTX (10). In CIA model, it can suppress the expression of VEGF in synovium (11) and reduce the local inflammation and the pannus formation. BZXD can reduce IL-1β level and relieve the arthritis which might result from regulating the immunity function, suppressing the TNF-α expression or combining with the TNF-α receptor and then interrupt the cytokine net. Whether the BZXD can block the cartilage destruction or not, there still has a lot of work to do.
